# Ectopic Filtering Bleb After Ahmed Glaucoma Valve Implantation Treated With Transconjunctival Mattress Sutures: A Case Report

**DOI:** 10.7759/cureus.108466

**Published:** 2026-05-08

**Authors:** Tommaso Bonifazi, Takayuki Naito, Chisako Ida, Hinako Ohtani, Keigo Takagi, Yuto Yoshida, Masaki Tanito

**Affiliations:** 1 Department of Ophthalmology, Shimane University Faculty of Medicine, Izumo, JPN

**Keywords:** ahmed glaucoma valve implantation, bleb revision, ectopic bleb, giant bleb, glaucoma surgery

## Abstract

Giant bleb formation is an uncommon complication following Ahmed glaucoma valve implantation and has most often been described in association with localized encapsulation around the valve plate. We report the case of a 74-year-old man with pseudoexfoliation glaucoma who developed a large cystic inferonasal bleb six weeks after pars plana implantation of an Ahmed glaucoma valve in the inferotemporal quadrant of the right eye. At presentation, the intraocular pressure was 7 mmHg, and slit-lamp examination suggested communication between the base of the bleb and the valve plate. The bleb was drained, and two transconjunctival horizontal mattress sutures were placed at the presumed site of communication. Following surgery, the ectopic bleb regressed completely, and intraocular pressure remained stable throughout follow-up for 10 months. This case suggests that ectopic bleb formation may arise through mechanisms other than encapsulation, likely related to redistribution of aqueous humor along adjacent subconjunctival tissue planes. In such cases, transconjunctival suturing may represent an effective approach to limit bleb expansion while preserving implant function.

## Introduction

Glaucoma drainage devices (GDDs), including the Ahmed glaucoma valve (AGV), are widely used in the management of refractory glaucoma. Their effectiveness relies on the formation of a filtering bleb over the endplate, whose morphology and behavior depend on the interaction between the device, aqueous outflow, and surrounding tissues [1]. Postoperative complications are largely related to tissue response and wound healing, and include fibrosis, encapsulation, and device exposure [1,2].

The development of an enlarged or “giant” filtering bleb is an uncommon complication that may lead to ocular surface disturbances, discomfort, and cosmetic deformity [2,3]. In most reported cases, giant bleb formation is related to localized encapsulation around the valve plate, resulting in enlargement in continuity with the implant [4,5]. More rarely, bleb enlargement has been attributed to redistribution of aqueous humor through direct fistulous pathways [6].

It has been reported that giant blebs can be classified into the anterior enlargement type, which extends anterior to the anterior edge of the plate, and the posterior enlargement type, which enlarges posterior to the posterior edge of the plate, in order to determine the treatment strategy [2,3]. However, cases showing bleb extension beyond the plate may not fit into this classification. We report a case of ectopic giant bleb formation in the inferonasal quadrant following inferotemporal implantation of an AGV, in which communication between the base of the ectopic bleb and the valve plate was observed. The ectopic bleb was successfully treated with drainage and transconjunctival horizontal mattress sutures placed at the site of communication.

## Case presentation

A 74-year-old Japanese man with pseudoexfoliation glaucoma in the right eye (OD) and ocular hypertension in the left eye (OS) was referred for surgery because of inadequate intraocular pressure (IOP) control despite maximal tolerated medical therapy with latanoprost in both eyes (OU) and a fixed combination of dorzolamide/timolol in OD. At presentation, best-corrected visual acuity (BCVA) was 20/25 OD and 20/20 OS, and IOP measured by Goldmann applanation tonometry (GAT) was 26 mmHg OD and 14 mmHg OS. Fundus examination revealed advanced glaucomatous optic nerve damage OD, with a vertical cup-to-disc ratio of 0.9 and marked neuroretinal rim pallor, whereas OS showed moderate cupping (0.6). The mean deviation (MD) on the Humphrey 30-2 visual field test (Humphrey Field Analyzer, Carl Zeiss Meditec, Dublin, CA, USA) was −27.8 dB OD and −0.8 dB OS. Slit-lamp examination showed conjunctival hyperemia, mild punctate epithelial keratopathy, and pseudoexfoliative material on the surface of the intraocular lens OD. Corneal endothelial cell density (CECD) was 2,568 cells/mm² OD and 2,372 cells/mm² OS (EM-3000, Tomey, Nagoya, Japan). Posterior vitreous detachment was present OU, and aqueous flare was 28.0 ± 0.4 pc/ms (FM-600α, Kowa, Tokyo, Japan) OD. His past ocular history included uncomplicated cataract surgery in OU.

Given the presence of thin conjunctiva and Tenon’s capsule, increased aqueous flare, abundant pseudoexfoliative material [7], and posterior vitreous detachment, a 25-gauge pars plana vitrectomy combined with AGV-FP7 implantation (New World Medical Inc., Rancho Cucamonga, CA, USA) was performed in the superotemporal quadrant of OD in June 2022 using a micro-incision scleral tunnel (MIST) technique [8]. A similar procedure was performed in OS in May 2023. After two years of adequate IOP control, the initial procedure in OD failed due to bleb fibrosis, and in May 2025, a second AGV-FP7 implantation was performed in the inferotemporal quadrant via a pars plana approach. Postoperatively, topical levofloxacin and a corticosteroid were administered four times daily for four weeks. The early postoperative course was uneventful.

**Figure 1:**
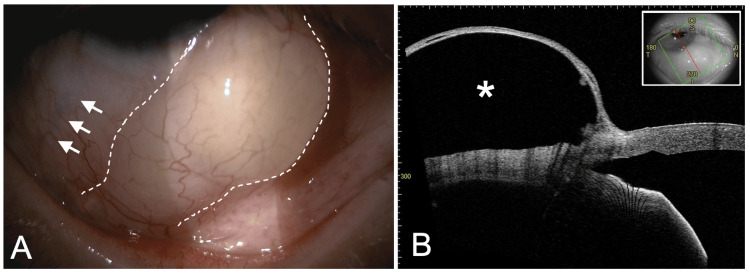
Preoperative findings (6 weeks after the Ahmed implantation). (A) Slit-lamp photograph of the right eye showing a large cystic bleb in the inferonasal quadrant (dashed line). The course of the tube from the inferotemporal quadrant toward its pars plana entry site is indicated by arrows. (B) Anterior segment optical coherence tomography (AS-OCT) demonstrating a well-defined hyporeflective cavity corresponding to the ectopic bleb (*). The figure was assembled by the authors using Microsoft PowerPoint (Microsoft Corp., Redmond, WA, USA).

Approximately six weeks after surgery, the patient noted a conjunctival elevation in OD. IOP was 7 mmHg, and the patient reported minimal symptoms, mainly related to cosmetic appearance. Slit-lamp examination revealed a large inferonasal bleb with apparent communication with the bleb over the inferotemporal implant plate (Figure 1A). Anterior segment optical coherence tomography (AS-OCT; Tomey Corporation, Nagoya, Japan) confirmed a well-defined hyporeflective cavity corresponding to the ectopic bleb (Figure 1B). The bleb showed no spatial relationship to the tube or its entry site. Given the absence of significant symptoms, observation was initially chosen; however, progressive enlargement prompted surgical revision.

**Figure 2:**
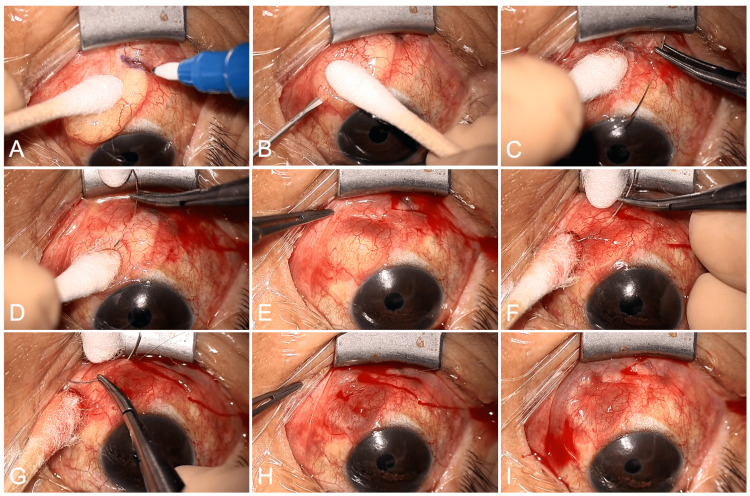
Transconjunctival horizontal mattress suturing for ectopic bleb reduction. The communication site between the ectopic bleb and the bleb over the implant plate is identified and marked on the conjunctiva (A). A small incision is then made in the bleb wall to allow drainage of its contents (B). A 10-0 polyester suture is introduced through the conjunctiva (C), passed in a parallel course in the opposite direction (D), and gently tightened (E). A second pass is performed adjacent to the initial tract (F, G), and the suture is secured (H). Final appearance at the end of the procedure (I).

**Figure 3:**
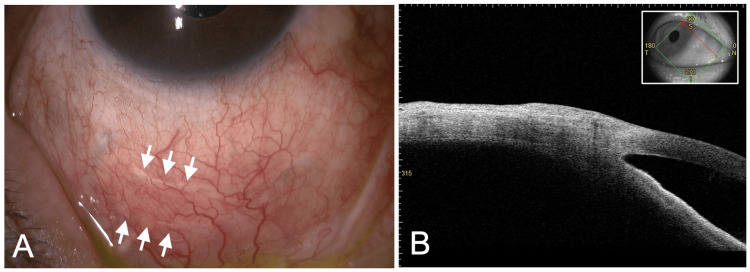
Postoperative findings 2 months after ectopic bleb reduction. (A) Slit-lamp photograph showing complete resolution of the inferonasal ectopic bleb after transconjunctival horizontal mattress suturing. The sutures are visible beneath the conjunctiva (arrows). (B) Anterior segment optical coherence tomography (AS-OCT) demonstrating collapse of the cavity. The figure was assembled by the authors using Microsoft PowerPoint (Microsoft Corp., Redmond, WA, USA).

After a small incision was made to drain the bleb contents (Figure 2), two transconjunctival horizontal mattress sutures were placed using a 10-0 polyester suture and a PC-9 needle (Alcon Laboratories, Fort Worth, TX, USA) at the presumed site of communication between the ectopic bleb and the bleb over the implant plate (Video 1). Postoperatively, topical levofloxacin and a corticosteroid were administered for two weeks. In the immediate postoperative period, IOP was 11 mmHg, with a marked reduction in bleb size and a subconjunctival hemorrhage in the inferior quadrants. At two months, the ectopic bleb had completely regressed (Figure 3), the sutures placed on the conjunctiva spontaneously became buried beneath the conjunctiva (Figure 3), and IOP remained within the target range. At the 10-month follow-up, IOP was below 10 mmHg without topical therapy, and no additional intervention was required.

**Video 1:** Intraoperative video showing drainage of the ectopic bleb and placement of transconjunctival horizontal mattress sutures.

## Discussion

Giant bleb formation following GDD implantation is an uncommon complication that may lead to discomfort, foreign body sensation, exposure-related keratitis, proptosis, and cosmetic concerns [2,3]. In most cases, bleb enlargement results from localized encapsulation around the implant plate and remains in direct continuity with the surgical site [2-5]. Ugarte et al. classified giant blebs after AGV implantation into anterior and posterior types based on the direction of expansion relative to the implant plate [3]. However, this classification does not account for cases in which bleb enlargement occurs outside the quadrant of implantation. Younger and Kooner [6] described extension of aqueous humor into the eyelid through a fistulous pathway, suggesting that mechanisms other than localized encapsulation may contribute to bleb enlargement.

In our case, the implant was located in the inferotemporal quadrant, whereas a well-defined giant bleb developed in the inferonasal quadrant. Slit-lamp examination suggested communication between the base of the ectopic bleb and the plate. The occurrence of the bleb in a different quadrant, together with its limited continuity with the implant, does not fit the typical pattern of encapsulation-related enlargement and instead is more consistent with redistribution of aqueous humor along adjacent tissue planes. The tube had been inserted using the MIST technique [8], which employs a scleral tunnel for fixation. Although the MIST technique could theoretically contribute to altered tissue planes and facilitate ectopic bleb formation, this appears unlikely in the present case, as the bleb appeared to communicate with the plate and showed no spatial relationship to the scleral tunnel or tube track (Figure 1).

Surgical management of giant blebs usually involves bleb wall recession or excision, both requiring conjunctival dissection and direct manipulation of the bleb capsule [2,3]. While effective, these approaches may not be required when bleb enlargement is not primarily related to encapsulation. Transconjunctival compression sutures have long been used in filtering surgery to manage overfiltration and hypotony by increasing resistance and reducing bleb height [9-11]. First described by Shirato et al. for hypotony maculopathy [9], this technique has been adopted as a less invasive alternative to surgical revision in selected cases [10].

This case highlights a rare pattern of giant bleb formation, likely related to aqueous humor redistribution through altered tissue planes. After drainage of the ectopic bleb, transconjunctival horizontal mattress sutures were placed at the suspected site of communication between the ectopic bleb and the implant plate. This resulted in complete regression of the ectopic bleb, with stable IOP and preservation of a functioning bleb over the plate. Because the sutures spontaneously became buried beneath the conjunctiva, suture removal was also unnecessary. Giant bleb enlargement may arise from distinct mechanisms, including localized encapsulation and altered aqueous redistribution. In the latter case, treatment should be directed at modifying aqueous flow pathways.

## Conclusions

Giant bleb formation after GDD implantation may not always be attributable solely to localized encapsulation around the implant plate. This case suggests that ectopic giant bleb formation following AGV implantation may be related to the redistribution of aqueous humor along adjacent subconjunctival tissue planes. In the present case, surgical treatment aimed at interrupting communication with the valve plate using transconjunctival mattress sutures was associated with complete regression of the ectopic bleb while preserving implant function. Although this is a single case report and causality cannot be definitively established, the findings may help generate hypotheses regarding the mechanisms and management of atypical giant bleb formation after GDD implantation.
